# Increase in TGF-β Secreting CD4^+^CD25^+^ FOXP3^+^ T Regulatory Cells in Anergic Lepromatous Leprosy Patients

**DOI:** 10.1371/journal.pntd.0002639

**Published:** 2014-01-16

**Authors:** Chaman Saini, Venkatesh Ramesh, Indira Nath

**Affiliations:** 1 National Institute of Pathology, Safdarjung Hospital Campus, New Delhi, India; 2 Department of Dermatology, Safdarjung Hospital, New Delhi, India; Swiss Tropical and Public Health Institute, Switzerland

## Abstract

**Background:**

Lepromatous leprosy caused by *Mycobacterium leprae* is associated with antigen specific T cell unresponsiveness/anergy whose underlying mechanisms are not fully defined. We investigated the role of CD25^+^FOXP3^+^ regulatory T cells in both skin lesions and *M.leprae* stimulated PBMC cultures of 28 each of freshly diagnosed patients with borderline tuberculoid (BT) and lepromatous leprosy (LL) as well as 7 healthy household contacts of leprosy patients and 4 normal skin samples.

**Methodology/Principle Findings:**

Quantitative reverse transcribed PCR (qPCR), immuno-histochemistry/flowcytometry and ELISA were used respectively for gene expression, phenotype characterization and cytokine levels in PBMC culture supernatants. Both skin lesions as well as *in vitro* antigen stimulated PBMC showed increased percentage/mean fluorescence intensity of cells and higher gene expression for FOXP3^+^, TGF-β in lepromatous (p<0.01) as compared to tuberculoid leprosy patients. CD4^+^CD25^+^FOXP3^+^ T cells (Tregs) were increased in unstimulated basal cultures (p<0.0003) and showed further increase in *in vitro* antigen but not mitogen (phytohemaglutinin) stimulated PBMC (iTreg) in lepromatous as compared to tuberculoid leprosy patients (p<0.002). iTregs of lepromatous patients showed intracellular TGF-β which was further confirmed by increase in TGF-β in culture supernatants (p<0.003). Furthermore, TGF-β in iTreg cells was associated with phosphorylation of STAT5A. TGF-β was seen in CD25^+^ cells of the CD4^+^ but not that of CD8^+^ T cell lineage in leprosy patients. iTregs did not show intracellular IFN-γ or IL-17 in lepromatous leprosy patients.

**Conclusions/Significance:**

Our results indicate that FOXP3^+^ iTregs with TGF-β may down regulate T cell responses leading to the antigen specific anergy associated with lepromatous leprosy.

## Author Summary

Lepromatous leprosy is a generalized infectious disease caused by *Mycobacterium leprae* with the patients showing T cell mediated unresponsiveness to the pathogen and chronicity of lesions. The causation of unresponsiveness and anergy in this form of leprosy is not fully understood. The recent discovery of CD25^+^FOXP3^+^ cells with regulatory functions (Tregs) in mice and man have made it possible to study their role in the dampening of T cell responses in lepromatous leprosy. We investigated both skin and PBMC from leprosy patients for lineage specific molecular, and phenotypic markers of Tregs as well as cytokines *in situ* and in *in vitro M.leprae* stimulated PBMC cultures (iTreg). Our studies find an increase in lineage specific CD4^+^ iTregs in lepromatous leprosy as compared to the limited form of borderline tuberculoid leprosy. Such cells secrete TGF-β, an inhibitory cytokine and may play a role in negatively regulating the T cell immune responses in lepromatous disease.

## Introduction

The hall mark of leprosy caused by *Mycobacterium leprae* is involvement of the skin and peripheral nerves of man. Leprosy patients present with varied clinic-pathological manifestations and bacterial load which are influenced by the host immune response. Tuberculoid leprosy, both polar (TT) and borderline forms (BT) show localized paucibacillary, hypo pigmented, hypo anesthetic patches and early nerve damage. Borderline (BL) and polar forms of lepromatous leprosy (LL) present as generalized disease with multiple, multibacillary skin patches along with involvement of other organs [1]. Whereas tuberculoid leprosy patients show good recall T cell mediated immune and poor antibody responses to the *M.leprae* antigens, lepromatous patients show a reverse pattern. Moreover, LL patients show specific T cell unresponsiveness to the causative organism though capable of mounting T cell responses to other antigens. The mechanisms underlying the antigen specific anergy are being intensely investigated. In the 70 s a subset of suppressor T cells were first described as a distinct population that inhibited responses through soluble factors [2]. Such cells with CD8 phenotype were thought to be responsible for the T cell anergy in lepromatous leprosy [3]. Others found that monocytes/macrophages from LL patients, either alone [4] or through soluble factors including PGE2 and IL-10 were able to suppress *in vitro* T cell responses [5], [6]. Suppressor T cells became a subject of controversy amongst immunologists as molecular or biochemical markers could not be found to identify this subset. With the discovery of Th1 and Th2 subsets having mutually exclusive cytokine patterns, suppression was thought to be mediated by regulatory cytokines [7]. Th1 and Th2 were reported to be associated with tuberculoid and lepromatous leprosy respectively and became a popular notion to explain the leprosy spectrum [8]. However, the finding that some patients of both clinical types of leprosy also showed non polarized Th0 subset with production of both IFN-γ and IL-4 was intriguing and made it difficult to reconcile the leprosy spectrum and anergy based solely on the Th1 and Th2 paradigm [9]. Recent studies from our laboratory showed that leprosy patients with the non polarized Th0 subset had increased percentage of Th17 cells which may constitute the third subset of Th types in leprosy patients who failed to show Th1 and Th2 polarization [10]. Nevertheless, the nature of antigen specific anergy in leprosy continues to evade consensus.

A seminal discovery was made in 1995 by Sakaguchi et al [11] who showed that cells responsible for inhibition of organ specific autoimmunity were CD4^+^ T cells which expressed CD25 and later shown to also express transcription factor forkhead box P3 (FoxP3) in mice [12] and man [13]. The discovery of CD4^+^CD25^+^FOXP3^+^ cells as suppressors of autoimmune responses and their presence in man [14] has revived the concept of a distinct lineage of T cells that negatively regulate the immune responses in order to maintain homeostasis and were designated as regulatory T cells (Tregs). Though various subsets of Tregs have been described, in general they can be divided into thymus derived naturally occurring Tregs and peripherally derived adaptive/inducible Tregs (Th3, Tr1) [15]. Whereas the former act by direct cell contact, the latter induce suppression through cytokines TGF-β (Th3) and IL-10 (Tr1) [15], . FOXP3 is thought to be a primary requirement for suppressive function. However, in the humans, low expression has been noted transiently in CD4^+^CD25^neg^ non suppressive T cells and activated T cells with and without [17], [18] suppressive function. Moreover, in the periphery, CD4^+^CD25^+^ Tregs may be induced by antigen from CD4^+^CD25^neg^ naïve T cells. Though mice express CD25 constitutively, in the human only Tregs that highly express it (CD25^hi^) show suppressive activity [16]. Other human subtypes that have been suggested include CD45RA^+^FOXP3^low^, CD45RA^−^FOXP3^+^ resting and activated Tregs respectively as well as CD45RA^neg^ FOXP3^low^ non suppressive, cytokine secreting Tregs [19]. Recently, recommendations have been made to simplify the nomenclature of Treg cells to include ‘thymus derived’(nTreg) instead of ‘natural’ and ‘peripheral derived’ instead of ‘induced or adaptive’ and that ‘*in vitro*-induced Tregs (iTregs) should be distinguished from populations generated *in vivo* [20]. .

With a view to understanding the antigen specific anergy associated with the generalized form of leprosy, we revisited the concept of suppressor T cells by investigating the role of Tregs in skin lesions and *in vitro* stimulated PBMC cultures of lepromatous and tuberculoid leprosy patients as well as healthy household contacts using qPCR for gene expression, flowcytometry for phenotype characterization and ELISA for cytokine levels in culture supernatants. Though we await the acceptance of the new nomenclature, we thought it appropriate to use iTreg to denote *in vitro* stimulated PBMC in this study.

Taken together, our studies show an increase in TGF-β^+^ CD4^+^ CD25^+^ FOXP3^+^ T cells both in dermal lesions and in *in vitro* induced antigen but not mitogen stimulated PBMC (iTreg) of lepromatous leprosy patients.

## Materials and Methods

### Experimental Strategy and Rationale

Amongst the leprosy types, lepromatous leprosy is associated with antigen specific T cell unresponsiveness/anergy whose underlying mechanisms have not been fully characterized. In view of recent reports of T regulatory (Treg) cells that dampen immune responses [16], we investigated the role of CD25^+^FOXP3^+^ T cells in patients with anergic generalized form of lepromatous leprosy and compared with patients of the more limited form of borderline tuberculoid leprosy (BT). Healthy house hold subjects with long time exposure to infected lepromatous leprosy patients and skin samples from healthy patients undergoing cosmetic surgery were included as controls. Skin lesions and mitogen/antigen stimulated PBMC were studied using quantitative reverse transcribed PCR (qPCR) for expression of genes along the *FOXP3* pathway, cytokines, transcription factors and signaling molecules; flowcytometry was used for identification of cell types and fluorescence intensity of markers in PBMC and ELISA for measuring cytokines TGF-β, IL-10, IFN-γ and IL-17 in the culture supernatants of stimulated PBMC.

### Patients and Controls

A total of 56 newly diagnosed untreated leprosy patients (43 males, 13 females aged between 19–60 years) from Leprosy Clinics of the Department of Dermatology, Safdarjung Hospital, New Delhi were included in the study (Table 1) and classified on the basis of Ridley-Jopling classification [1]. Study group included 28 borderline tuberculoid (BT), 28 lepromatous (LL), 7 healthy household contacts (HC) exposed to leprosy patients and skin samples from 4 healthy subjects undergoing cosmetic surgery. Exclusion criteria included patients below 15 years of age, pregnant women, clinical evidence of anemia and other infections such as tuberculosis, HIV and helminthic infestation. Skin biopsies from 10 each of BT, LL patients and 4 normal subjects were investigated for immunohistochemistry and gene expression studies by quantitative RT-PCR (qPCR). PBMC were investigated on additional 10 each of BT, and LL patients for gene expression; other 8 each of BT and LL subjects, for flow cytometry analysis.

**Table 1:** Clinical details of 56 newly diagnosed untreated leprosy patients and 11 healthy control subjects.

Clinical types	Number of Patients	Sex		Age	BI	Duration of leprosy
		M	F	(years)		(months)
Borderline Tuberculoid (BT)	28	22	06	20–57	0–0.5	1–30
Lepromatous Leprosy (LL)	28	21	07	19–60	4.5–6	6–14
Healthy contacts (HC)	07	05	02	22–40	-	-
Normal Skin(N)	04	03	01	22–28	-	-

Patients were typed on the basis of Ridley Jopling classification [1], BI; Bacillary Index (mean of six lesional sites). M; male, F; female. BT: Borderline Tuberculoid, LL: Lepromatous Leprosy, HC: Healthy house hold contacts with long exposure to leprosy patients. N: Normal skin samples from subjects undergoing cosmetic surgery.

### Ethics Statement

The study was approved by Institutional Ethical Committee [08-09-EC (3/7)] of Safdarjung Hospital, New Delhi, India. Informed written consent was obtained from the patients after counseling and prior to obtaining blood and tissue samples.

### PBMC Isolation and *In Vitro* Cultures

Fresh PBMC were isolated in <2 h after obtaining the sample, from 10 ml of sterile heparinized (Brawn laboratories, Haryana India) blood by Ficoll-Hypaque density gradient method (Histopaque, Sigma Aldrich, USA) after diluting with 1∶1 in RPMI 1640 (Sigma Aldrich, MO, USA) as described earlier [10]. In brief mononuclear cells were isolated by centrifugation at 800 g for 20 minutes, washed three times in sterile 1× HBSS (GIBCO, NY, USA) and re-suspended in RPMI 1640 with 10% pooled human AB serum, 2 mM L-glutamine, 100 units of penicillin (Alembic Chemicals, India) and 100 µg streptomycin (Sarabhai Chemicals, India)). Cell yield ranged from 1.3 to 1.5×10^6^ per ml and cell viability ranged from 95–98% as estimated by 0.2% trypan blue staining (Sigma Aldrich, MO, USA) 2×10^6^ cells/ml were cultured for 48 h in sterile flat bottom 24- well plates (Falcon, NJ, USA) with and without 25 µl of T cell mitogen PHA (5 µg/ml of phytohemagglutinin, Sigma) and of heat killed armadillo derived *M leprae* sonicated antigen (10 µg/ml) kindly provided by P J Brennan of Colorado State University and incubated at 37°C in humidified 5% CO_2_+air. After harvest cells were washed as above and stored in RNA later (Sigma) for gene expression studies or immediately processed for flow cytometry analysis as given below. Paired culture supernatants were collected, centrifuged to remove cell debris and stored at −80°C for estimation of cytokines by ELISA.

### Skin Biopsies

Skin biopsies were obtained from typical lesions by anesthetizing the area with 1% lignocaine (Kremoint Pharma, Mumbai Maharashtra, India) and applying sterile 4 mm punch (Cardiograph Co, Satara, Maharashtra, India). Normal skin was obtained from 4 subjects undergoing cosmetic surgery. Part of the biopsy was processed in buffered formalin for routine histopathology and immunohistochemistry. The remainder was placed in 1 ml of RNA later (Sigma) and stored at −80°C till further use.

### RNA Isolation

RNA was isolated from: i) stored skin biopsies after thawing and crushing the tissue with liquid nitrogen in pestle and mortar, ii) PBMC were homogenized in 1 ml syringes using RNeasy Mini Kit (Qiagen, Maryland, USA) according to the manufacturer's instructions. The isolated RNA was quantified using Nanodrop spectrophotometer (Nanodrop Technologies, Wilmington, USA). Only samples with OD of 1.8 to 2.0 at 260/280 nm were used. The quality of RNA was also checked for 28 s and 18 s RNA by electropherogram using Bio analyzer (Agilent Technologies, Inc, Singapore). RNA Integration Number value of ≥7 was considered to be optimum.

### Reverse Transcriptase PCR Reaction (RT-PCR) and Real Time PCR (qPCR)

For cDNA synthesis 1 µg total RNA was transcribed with RT First strand kit (SA Biosciences, MD,USA). Reactions were performed according to the manufacturer's instructions and the cDNA stored at −20°C till further use. Gene expression was measured in real-time using customized real time PCR arrays (SA Biosciences, Quiagen Co. CA, USA) as per the manufacturer's instructions. Duplicate samples of cDNA from antigen stimulated PBMC from each subject was amplified in 96 well plates containing primers for the genes of interest, cytokines IL-2, TGFβ, IL-10, IL-27 and IL-25, CD marker CD28, transcription factors FOXP3, STAT5A, GATA3, NFkB1, STAT3, STAT4 and chemokine IL-8 as well as 5 housekeeping genes β2M, HPRT1, RPL13A, GAPDH, ACTB. 1 µg of cDNA was used per reaction in wells containing the ready to use PCR master mix and appropriate primers. These were then subjected to qPCR (ABI 7000, Applied Biosystems Singapore) for 2 h. Threshold cycle values were normalized and expressed as ΔCt: mean Ct of gene of interest - mean Ct of set of 5 housekeeping genes.

### Flowcytometry

For intracellular staining, *in vitro* antigen and PHA stimulated cells were incubated with monensin (BD GolgiStop) for 8 h prior to harvest to block secretion of cytokine. All reagents were obtained from BD Biosciences, San Diego, CA. and used as per manufacturer's instructions. Staining was undertaken within 1 h after harvest and washing three times as above and determining cell viability which ranged from 91–95%. In brief, for cell surface staining, 0.5×10^6^cells/50 µl in staining buffer were incubated with a cocktail containing anti human CD3 (Per cpcy-5.5, clone:UCHT1), CD4 (APC-H7, clone:SK3), CD8 (PE-Cy7, clone:RPA-T8) and CD25 (FITC, clone:M-A251) for 45 min at 4°C. After cell surface staining, cells were incubated with 1× FOXP3 buffer A for 10 min at room temperature; cells were washed two times and permeabilized with buffer C for 30 min at room temperature. The cells were washed two times, resuspended in stain buffer and incubated with anti human FOXP3 (APC, clone:259D/C7) and TGF-β (PE, clone:TW4-9E7) at room temperature for 30 min in the dark, followed by two washes as before and resuspended in 500 µl. For evaluating phosphorylation of STAT5A (Alexa Flour-647, clone:47/stat5(PY694), cells were first fixed for 10 min at room temperature, permeabilized as before with appropriate buffer and stained with a cocktail of anti human STAT5A anti human CD25, CD3, CD4 and CD8 antibodies.. CD3^+^CD4^+^ and CD3^+^CD8^+^ T cells were gated following forward angle and side scatter characteristics of lymphocytes. Results were analyzed using BD FACS aria flow cytometry along with isotype controls of phycoerythrin (PE mouse IgG1), Alexa Fluor 488 (mouse IgG1), Alexa Flour 647 (mouseIgG1). Supplementary figure show strategy and standardization used for validating the results on multi color flowcytometry.

### Estimation of Cytokines by ELISA

Cytokines were estimated by ELISA (Ready Set Go, e-Bioscience, San Diego, CA, USA) as per manufacturer's instructions. In brief, 100 µl/well of cell free supernatants from antigen stimulated PBMC cultures were tested in duplicate in 96-well plates (Nunc, Rochester, NY, USA) pre-coated with biotin conjugated anti human antibodies for TGF-β IL-10, IFN-γ and IL-17. Plates were incubated overnight at 4°C, washed 5 times, blotted and wells blocked with assay diluents for 1 h at room temperature. After washing with buffer, appropriate avidin-horseradish peroxidase-conjugated anti-mouse antibody was added and the plates incubated at room temperature for 30 min. After washing as before, color development was undertaken using peroxidase color substrate TMB (Tetramethylbanzedine) and the reaction stopped by the addition of 1 N H_2_SO_4_. The optical density (OD) of each well was read at 450 nm.

### Immunohistochemistry (IHC)

4–5 µm thick formalin fixed paraffin embedded (FFPE) tissues were cut by rotary microtome (Leica Biosystems Nussloch, Germany), sections picked up on poly L-lysine (Sigma Aldrich, MO, USA) coated slides and stored at room temperature. Antibodies (dilution 1∶50) used in this study were mouse anti human FOXP3 (forkhead box protein3, e-Biosciences, San Diego, USA), rabbit polyclonal anti human TGF-β1 and IL-10, (Santa Cruz Biotechnology CA, USA). IHC was performed using enhancer HRP-polymer detection method (BioGenex, USA). In brief after deparaffinization, rehydration and blockade of endogenous peroxidase activity by 30% H_2_O_2_ and antigen-retrieval with Tris-EDTA (pH-9.0) buffer, sections were incubated with 1% albumin, bovine, pH 7.0 heat- shock fractionated protein block (USB co, Cleveland, OH USA) for 1 h, followed by incubation with anti-human FOXP3, TGF-β and IL-10 for 1 h. Color was developed using diaminobenzidine (DAB1) chromogen system. The staining protocols were all performed at room temperature except for the primary antibody incubation at 4°C in humidified chamber. Positive and negative stained cells were counted under the microscope using Image Pro express 6.0 software (Media cybernetics, USA) and percentage calculated after examining 1000 cells from multiple fields.

### Statistical Analysis

Nonparametric statistics was performed using Graph Pad Prism version 5 (Graph Pad Software, Inc., San Diego, CA, USA). Data were analyzed using two tailed Mann-Whitney test. p<0.05 was considered as statistically significant.

## Results

All subjects included in the study were evaluated clinically and by histopathological examination of skin biopsies using the Ridley Jopling classification [1]. Furthermore, immunological evidence of T cell responsiveness of the leprosy patients included in the present study for qPCR and flowcytometry was obtained by stimulating PBMC from 18 each of tuberculoid and lepromatous patients *in vitro* with antigens of armadillo derived leprosy bacilli for 48 h after optimizing time kinetics as given in Materials and Methods. Using interferon γ (IFN-γ) levels in culture supernatants as surrogate marker of T cell responsiveness it was noted that LL subjects were poorly responsive to *M.leprae* antigens *in vitro* as compared to BT patients (p<0.001, Figure S1A). IFN-γ was produced by both CD4^+^ and CD8^+^ T cells with higher percentage being observed with CD4^+^ lineage (Figure S1B) in conformity with our earlier reports [21], [22].

### Increase in FOXP3+, TGF-β+, IL-10+ Cells in Skin Lesions of Lepromatous Leprosy

**Figure 1:**
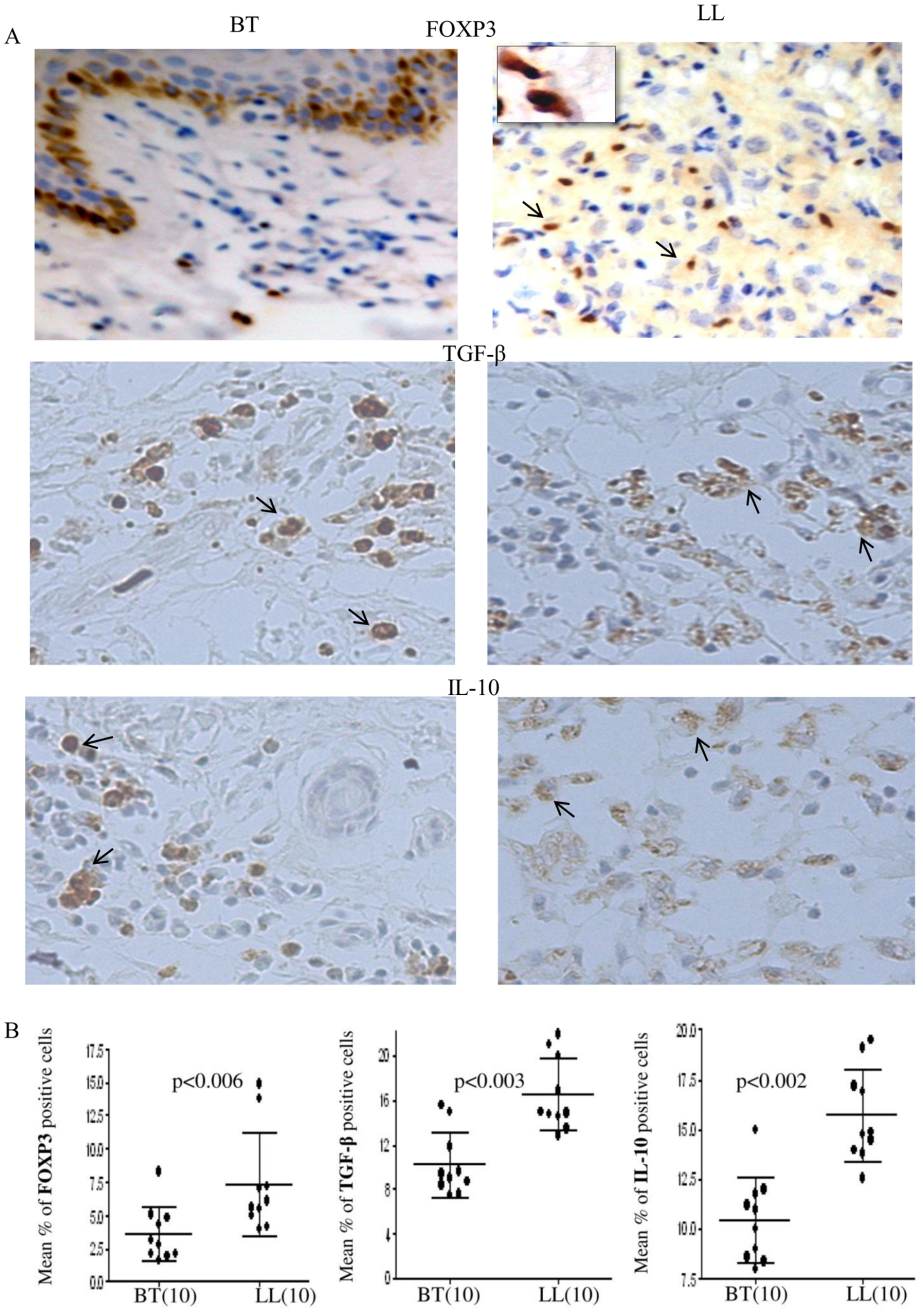
Increase in FOXP3^+^, TGF-β^+^ and IL-10^+^ cells in lepromatous leprosy skin lesions. Immunohistochemistry on representative skin lesions from borderline tuberculoid (BT) and lepromatous leprosy (LL) patients. **A**. FOXP3 with inset showing detail of nuclear staining, cytoplasmic TGF-β and IL-10 respectively. Diaminobenzidine was used as chromogen after treatment with appropriate anti-human antibodies as given in Materials and Methods. Original magnification: 200× inset 400× **B**. Scatter diagram showing increase of cells with FOXP3^+^ TGF-β^+^ and IL-10^+^ in lepromatous leprosy (LL) as compared to tuberculoid leprosy (BT) lesions. Horizontal and vertical bars indicate Mean%±SD of positive cells of 1000 total cells. p<0.05 was considered significant by two tail Mann Whitney test. Figures in parenthesis indicate the number of subjects.

Skin lesions of both tuberculoid and lepromatous leprosy patients showed the presence of nuclear FOXP3^+^ staining using immunohistochemistry (Figure 1). Whereas they were present in a circumscribed pattern around as well as amongst the epitheloid cells of the tuberculoid granulomas, FOXP3^+^ cells were scattered amongst the foamy macrophages of the lepromatous granulomas in the dermis. TGF-β and IL-10 showed diffuse cytoplasmic staining with the latter showing lower intensity in our hands. The distribution of positive cells for all markers was not uniform and 1000 cells were enumerated to obtain percentage of positive cells. As may be seen from Figure 1B there was significant increase in FOXP3^+^ cells (p<0. 006, two tailed Mann Whitney test) in lepromatous (LL) with Mean% ± SD of 7.3±3.8 as compared to 3.6±2.0 in tuberculoid leprosy (BT). TGF-β and IL-10 reported to be associated with FOXP3 cells [16] were seen in both leprosy types. The percentage of TGF-β and IL-10^+^ cells were also significantly higher in lepromatous (p<0.003, p<0.002 respectively) with Mean% ± SD being 16.55±3.2, as compared to 10.2±2.9 in tuberculoid leprosy granulomas.

**Figure 2:**
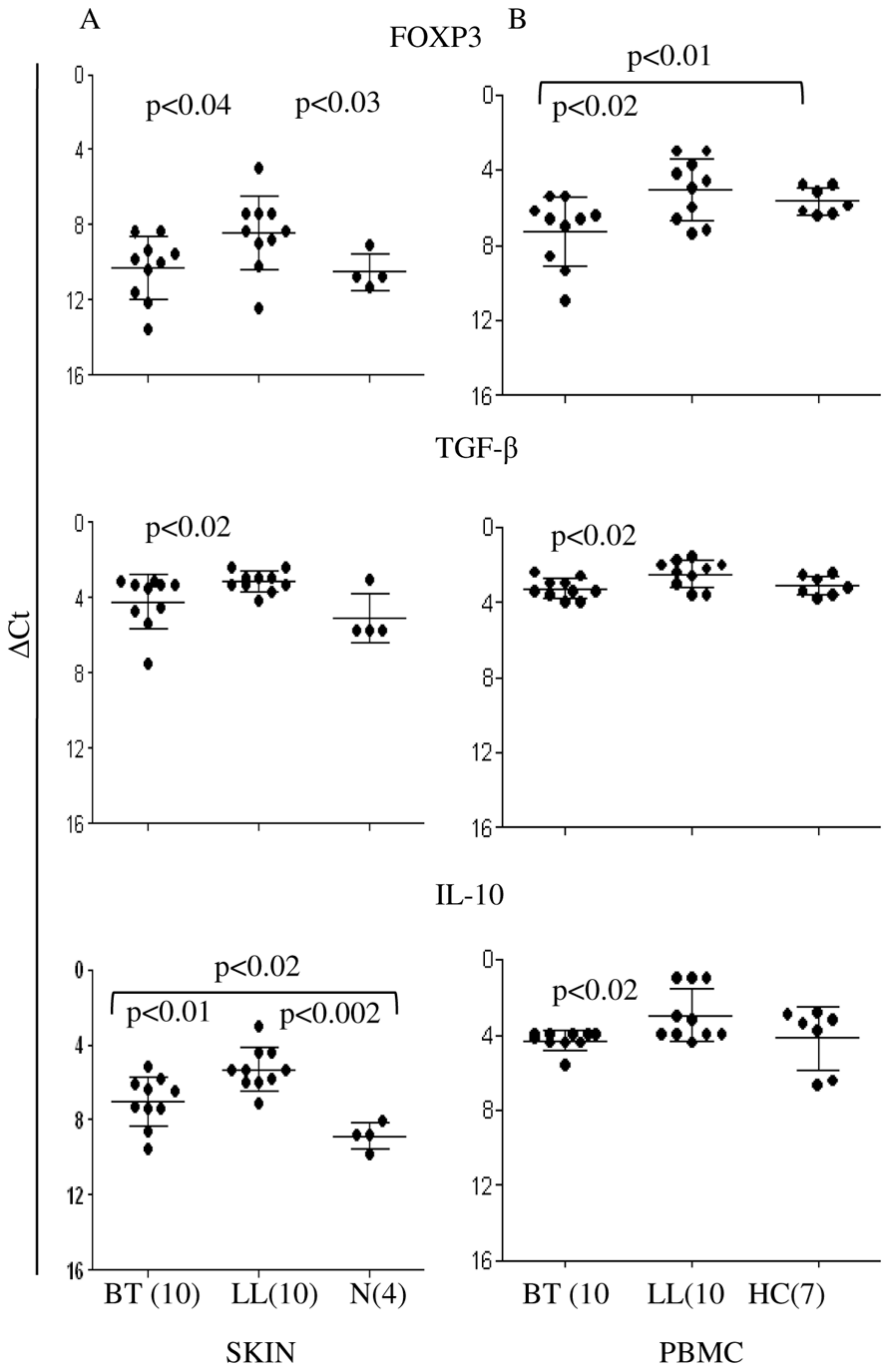
Increase in expression of FOXP3, TGF-β and IL-10 in lepromatous leprosy skin and armadillo derived heat killed *Mycobacterium leprae* (ML) stimulated *in vitro* PBMC cultures. Scatter diagram of gene expression (Mean ΔCt ± SD) in skin biopsies (A) and stimulated PBMC (B) of leprosy and healthy/normal subjects. Increase in gene expression was observed in lepromatous as compared to tuberculoid leprosy patients in both local sites and PBMC for FOXP3, TGF-β and IL-10. C. Abbreviations: LL: lepromatous leprosy, BT: borderline tuberculoid leprosy. N: skin from normal subjects undergoing cosmetic surgery. HC: healthy household contacts. Figures in parenthesis indicate number of subjects studied. p<0.05 was considered significant by two tailed Mann Whitney test.

In conformity with the above, using qPCR (Figure 2A) significant increase in gene expression was observed in lepromatous as compared to tuberculoid lesions and normal skin for FOXP3 (p<0.04 and p<0.03 respectively) TGF-β (p<0.02) and IL-10 (p<0.01 and p<0.002 respectively).

### Lepromatous Leprosy Patients Show Increase in FOXP3+ TGFβ Producing CD4+CD25+T Cells in Antigen Stimulated PBMC Cultures

In conformity with the findings observed in the skin, antigen stimulated PMBC (Figure 2B) showed higher expression of FOXP3, TGF-β and IL-10 in LL as compared to tuberculoid subjects (p<0.02). This was further confirmed by the increase of the cytokines in the PBMC culture supernatants by ELISA (Table 2 p<0.0003 and p<0.02 respectively for TGF-β and IL-10). Healthy contacts with long time exposure to leprosy patients also showed expression of FOXP3 and inhibitory cytokines to a lesser extent. In contrast, IFN-γ and IL-17 showed significantly higher levels in tuberculoid as compared to lepromatous leprosy (Table 2, p<0.01 and p<0.001 respectively).

**Table 2:** Mean pg/ml ± SD of cytokines in culture supernatants of 48 hr *M.leprae* stimulated PBMC from 10 each of tuberculoid (BT) and lepromatous leprosy (LL) patients investigated for gene expression studies.

Cytokines	Lowest detectable level (pg/ml)	pg/ml			
		BT		LL	
		Mean ± SD	[Range]	Mean ± SD	[Range]
**TGF-β**	8	105.4±46.7	[26.6–169.9]	335.9±108.1***	[163.0–577.6]
**IL-10**	2	60.4±25.6	[4.8–97.4]	119.1±75.8*	[54.2–299.0]
**IL-17**	30	101.9±26.28***	[45–169.7]	45.5±22.07	[0–111]
**IFN-γ** #	4	749.7±1314**	[58.4–4870]	53.12±23.3	[6–90]

Leprosy patients were typed as per Ridley Jopling classification [1].

p<0.01,

p<0.001,

p<0.0001 by two tailed Mann Whitney test.

P<0.05 was considered significant. Figures in parenthesis ( ) indicate number of patients, [ ] range in pg/ml. [0] below detectable level.

^#^ IFN-γ levels reflecting culture supernatants of patients investigated for both qPCR and Flow cytometry analysis is given in Figure S1.

**Figure 3:**
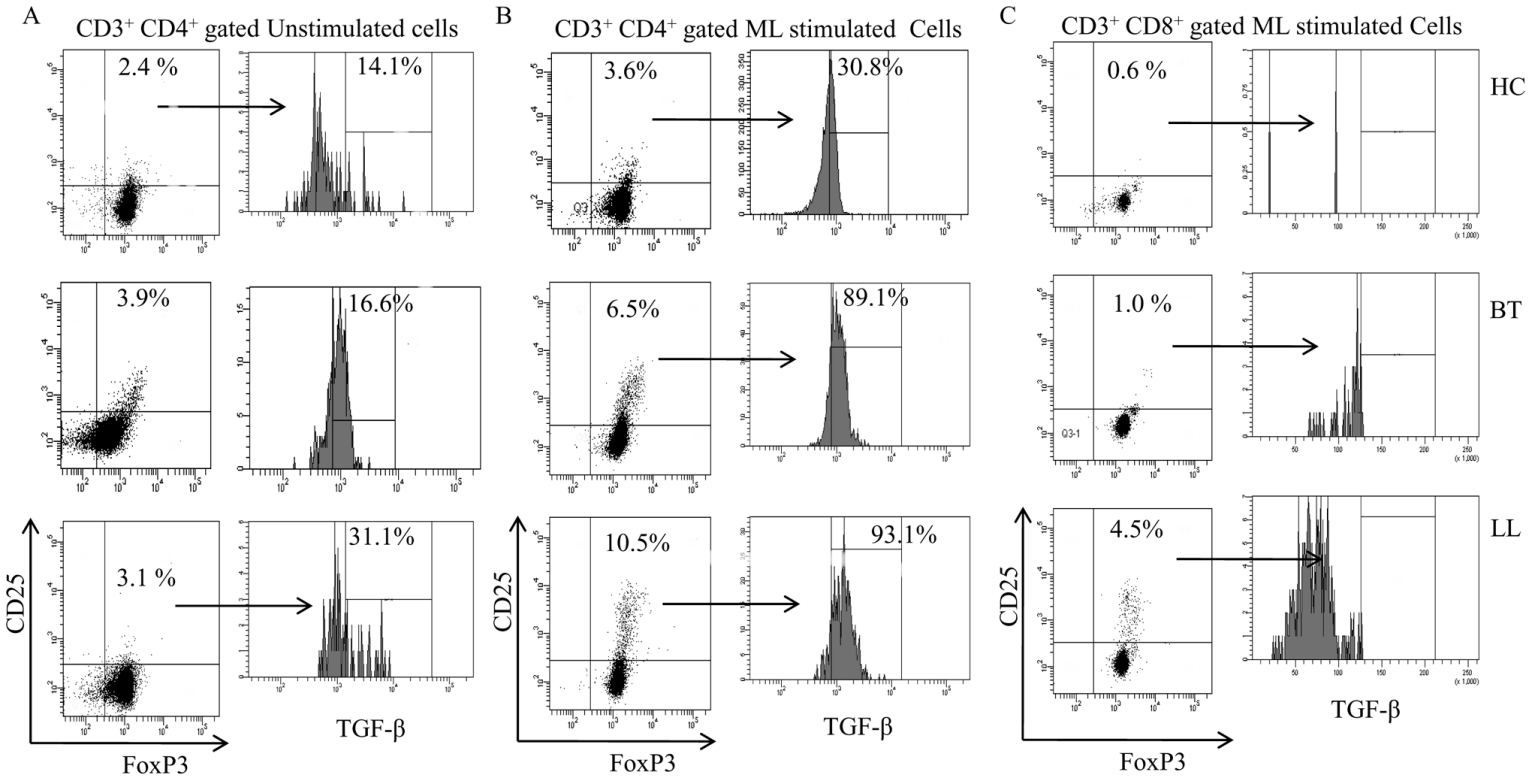
Flow cytometry analysis of PBMC cultures from representative leprosy patients and healthy subjects for FOXP3 and intracellular TGFβ. Unstimulated basal PBMC cultures (**A**) of lepromatous patients showed higher percentage of CD25^+^FOXP3+ T regs as compared to other clinical groups. *M. leprae* stimulated cultures (**B**) showed further increase in this population in lepromatous leprosy patients as compared to tuberculoid and healthy contacts. Increased percentage of TGF-β+ cells was observed in the Tregs of lepromatous as compared to other clinical groups in the basal unstimulated and antigen stimulated PBMC. CD8^+^ lineage of T cells (**C**) also showed increase in FOXP3 positivity in lepromatous leprosy. However, TGF-β was not detectable in these cells. Abbreviations and p values as in legend to Figure 2. iTreg: *in vitro* derived peripheral blood T cells with Treg signature markers of CD25 and FOXP3. Abbreviations as in Figure 2.

**Figure 4:**
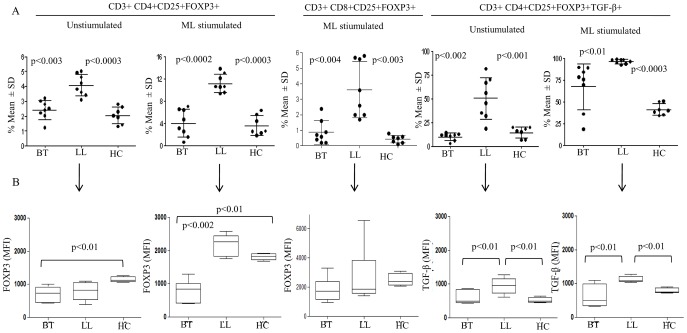
CD4^+^CD25^+^FOXP3^+^ iTreg cells are increased *in vitro* in PBMC of lepromatous patients and show intracellular TGF-β. **A**. Scatter diagram gives Mean% ± SD of FOXP3 cells from 48 hr PBMC cultures as given in Figure 4 and Materials and Methods. 10 each of lepromatous (LL), tuberculoid leprosy (BT) and 7 of healthy household contacts (HC) were investigated. CD3^+^, CD4^+^CD25^+^ cells showed significant increase in FOXP3 (iTregs) in lepromatous patients as compared to other clinical groups. **B**. This was further confirmed by mean fluorescence intensity (MFI) in all except unstimulated cultures for FOXP3 marker. Antigen stimulated PBMC showed increase in mean percentage as well as higher MFI with the lepromatous subjects showing higher values as compared to the other 2 groups. Intracellular TGF-β was associated with iTregs with significant increase in percentage of cells as well as in MFI in the lepromatous group. CD8^+^ lineage of cells also showed increase in FOXP3^+^ cells with negligible to low TGF-β (Figure 4). Abbreviations and p values as given in Figure 2.

To further characterize the nature of FOXP3+ cells we undertook flowcytometry analysis both in leprosy and house hold contact subjects. Figure S2 shows the strategy used for validating the antibodies and manual gating used in the study. Figure 3 shows both representative and group data on CD3+ gated cells in antigen stimulated PBMC cultures. Basal unstimulated PBMC gated for CD3+CD4+ showed low but significantly higher percentage but not mean fluorescence intensity (MFI) of CD25+FOXP3+ cells in lepromatous(p<0.003) as compared to other clinical groups (Figure 4) with Mean % ± SD being 4.0%±0.7 and 2.4%±0.6 respectively with contacts showing 2.0%±0.5. Antigen stimulated PBMC showed further increase in CD25^+^FOXP3^+^ cells in lepromatous subjects as compared to tuberculoid (p<0.0002) and healthy contacts (p<0.0003) indicative of increase in iTregs in the anergic form of leprosy. Moreover, CD25+FOXP3+ cells of basal cultures showing intracellular TGF-β was significantly higher in lepromatous as compared to tuberculoid leprosy (p<0.002). On antigen stimulation TGF-β^+^ cells increased further with lepromatous subjects, as expected showing significant increase in comparison to tuberculoid leprosy (p<0.01) and healthy subjects (p<0.0003). The Mean% ± SD of TGF-β producing cells in tuberculoid, lepromatous and healthy contacts was 67.4±26.1, 96.1±2.5, and 41.2±6.6 respectively (Figure 4A).

We further analyzed the data using Mean Fluorescence Intensity (MFI) of FOXP3 and TGFβ in the CD25^+^ T cell populations. Figure 4B confirms the above observations and shows the increase in MFI in lepromatous as compared to tuberculoid subjects for both FOXP3 (p<0.002) and TGF-β (p<0.01) in the CD4^+^CD25^+^ T cells in antigen stimulated PBMC cultures. That the iTreg discrimination noted in the two leprosy types was driven by *M.leprae* antigens was indicated by PHA stimulated PBMC cultures which showed general increase but not statistically significant differences between the two leprosy types either in percentage of cells with lineage specific markers or in MFI for FOXP3 and TGF-β (Figure S3).

CD8^+^ population of T cells with CD25^+^FOXP3^+^ were lower than the CD4^+^ T cells in both leprosy types and healthy contacts (Figures 3C, and 4). They were also significantly higher (Figure 4, p<0.004) in lepromatous (Mean% ± SD: 3.6±1.8) as compared to the tuberculoid (Mean% ± SD: 0.87±0.76) and healthy contact groups (Mean% ± SD: 0.43±0.24). Importantly, there was negligible intracellular TGF-β in CD8^+^ CD25^+^, FOXP3 cells in all 3 clinical groups which may reflect on their functional state (Figure 3C). Taken together the data provides evidence for increase in antigen induced iTregs in lepromatous leprosy which bear the signature markers of CD25 and FOXP3 in the CD4 lineage of T cells.

### Lepromatous Leprosy Shows Increase in FOXP3^+^ in CD25^hi^ and CD25^low^ Cells in Antigen Stimulated PBMC Cultures *In Vitro*

**Figure 5:**
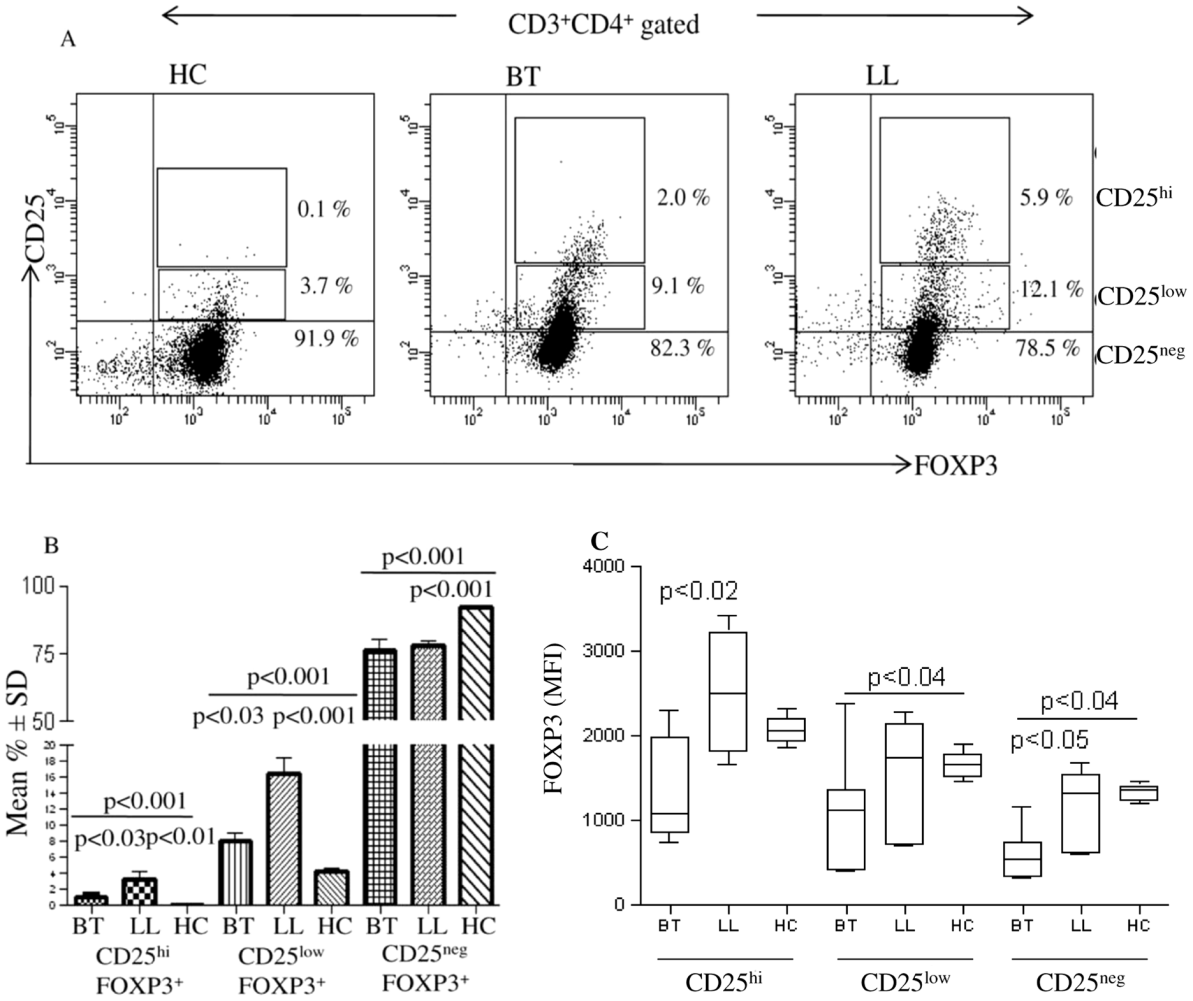
CD25^hi^ and CD25^low^ FOXP3^+^ iTreg cells show increase in lepromatous leprosy. **A**. Florescence intensity of CD25 was graded as high (hi) low and negative (neg) in CD3^+^CD4^+^ gated FOXP3^+^ cells. Representative flow cytometry analysis on 8 each of tuberculoid and lepromatous and 7 healthy subjects is shown in **B** where increase in percentage of CD25^hi^ was observed in *M. leprae* stimulated PBMC of lepromatous leprosy as compared to other clinical groups. **C**. Mean fluorescence intensity (MFI) of FOXP3 in the CD25 hi, CD25 low and CD 25^neg^ populations in the 3 clinical groups clinical groups but not. Abbreviations and p values as in legends to Figures 2 and 3.

We further graded the CD25 as high (hi), low and negative (neg) in FOXP3^+^ CD4^+^ T cells of antigen stimulated PBMC cultures. Figure 5A shows representative data of one subject each of the three clinical groups. As may be seen from Figure 5B, the percentage of CD25^hi^ was lower than the CD25^low^ population in both leprosy types. Importantly, significant increase in lepromatous as compared to tuberculoid and healthy subjects was observed with both the CD25^hi^ (p<0.03, p<0.01 respectively) and CD25^low^ (p<0.03, p<0.001 respectively) populations of FOXP3^+^ cells (Figures 5B,). Though CD25^neg^ FOXP3^+^ cells were present in higher percentages, they did not show discrimination between lepromatous and tuberculoid leprosy patients (Figure 5B). The MFI of FOXP3 also showed significant increase in lepromatous as compared to tuberculoid leprosy in both CD25^hi^ (p<0.02) and CD25^low^(p<0.04) population. However, in general MFI of FOXP3 was lower in the CD25^low^ populations in the leprosy groups as compared to the CD25^hi^ population. . Moreover, CD25^neg^ cells showed the lowest MFI in all three clinical groups (Figure 5C). Significant differences in MFI were observed between the clinical groups (p<0.05, p<0.04)), which needs further investigation as CD25^neg^ cells were reported to transiently express FOXP3 and be non suppressive in nature [17], [18].

#### Phosphorylation of STAT 5 accompanies TGF-β in Tregs

**Figure 6:**
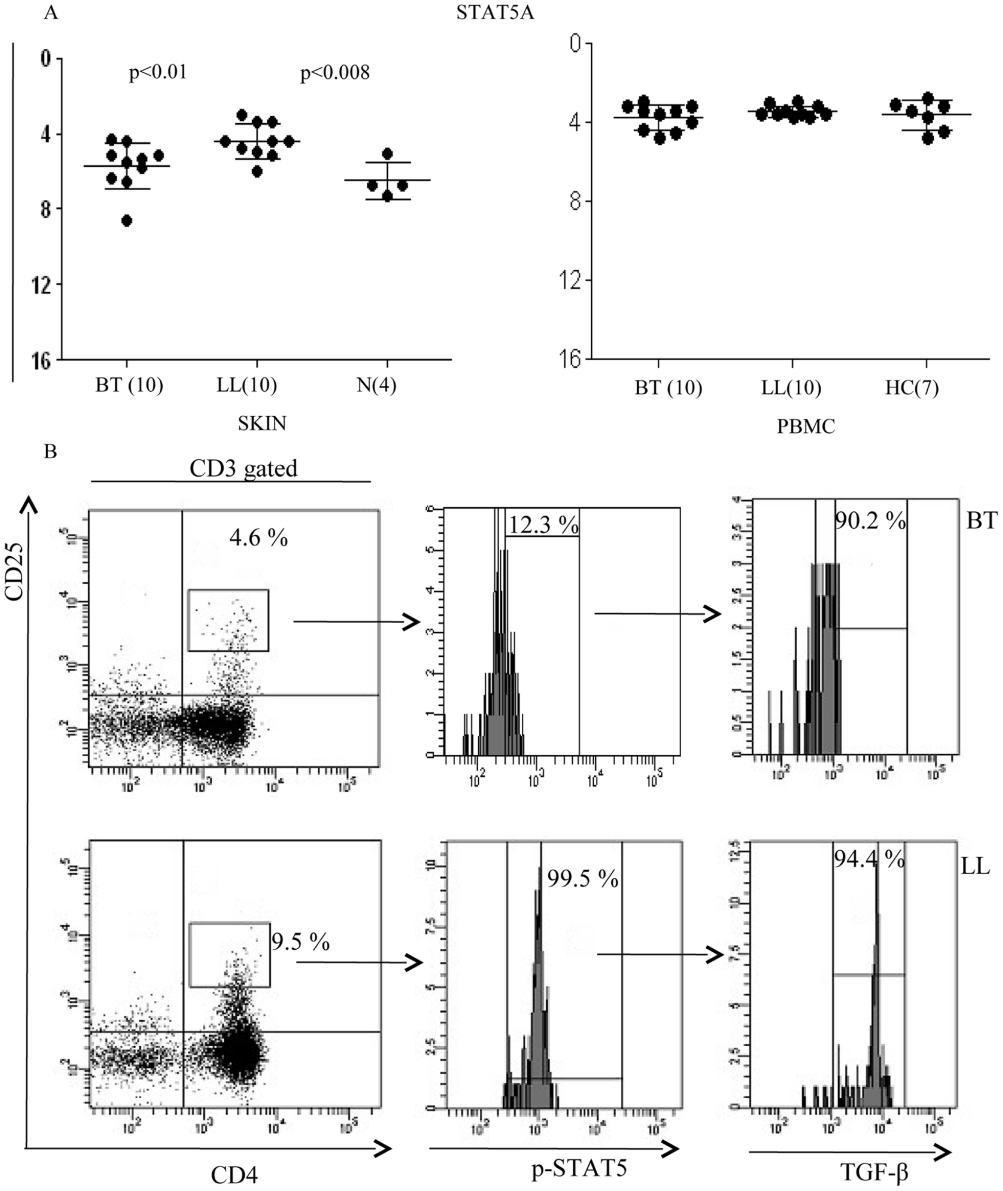
TGF-β presence in CD4^+^CD25^hi^ iTreg population is associated with phosphorylation of STAT5 (p-STAT5). **A**. Skin lesions and *M. leprae* stimulated PBMC of BT, LL and healthy subjects were investigated for expression of transcription factor STAT5A using quantitative reverse transcribed PCR (qPCR). Significant increase of STAT5 was observed in lepromatous as compared to tuberculoid leprosy lesions and normal skin. **B**. Representative flowcytometry analysis on antigen stimulated PBMC from 1 each of 3 BT and LL patients. The left panel shows the percentage of CD4+CD25+ double positive gated populations in one each of BT and LL patients and shows increase of this population in LL patient. The percentage of p-STAT5+ cells in the double positive populations are shown in the middle panel and again indicate the higher percentage in LL as compared to BT patients. The percentage of TGFβ+ cells that positive for STAT5 are given in the right panels and indicate that STAT5 was associated with TGFβ in both leprosy groups. Abbreviations and p values as in legends to Figures 2 and 3.

qPCR studies showed that expression of STAT5A was significantly higher in lepromatous skin in comparison to tuberculoid skin lesions (p<0.01) but did not show differences in antigen induced PBMC cultures of the leprosy types (Figure 6A). Therefore, we next investigated the status of STAT5A for TGF-β production in PBMC of 3 subjects each of both leprosy types using flowcytometry analysis. Figure 6B shows representative data wherein majority of CD4^+^CD25^hi^ cells showed phosphorylated STAT5A in lepromatous leprosy with Mean% ± SD of 92.83±5.79 as compared to lower numbers in tuberculoid leprosy (14.27%±1.73%) patients. Furthermore, >90% of TGF-β^+^ cells belonged to the p-STAT5^+^ population indicating the importance of phosphorylation of this transcription factor for inducing the inhibitory cytokine.

In summary the above data indicates that CD4^+^CD25^+^FOXP3^+^ T cells are present at a higher level in both skin lesions and antigen stimulated PBMC in the anergic form of lepromatous leprosy. CD4^+^ and not CD8^+^ population of T cells produced the associated inhibitory cytokine TGF-β in CD25^+^ FOXP3^+^ T cell populations. MFI of FOXP3 decreased sequentially in CD25^hi^ CD25^low^ and CD25^neg^ populations of all 3 clinical types and showed discrimination between the leprosy types. Phosphorylation of STAT5A appeared to be important for TGF-β production in these cells.

#### CD4^+^CD25^hi^ T cells do not show pro-inflammatory cytokines

**Figure 7:**
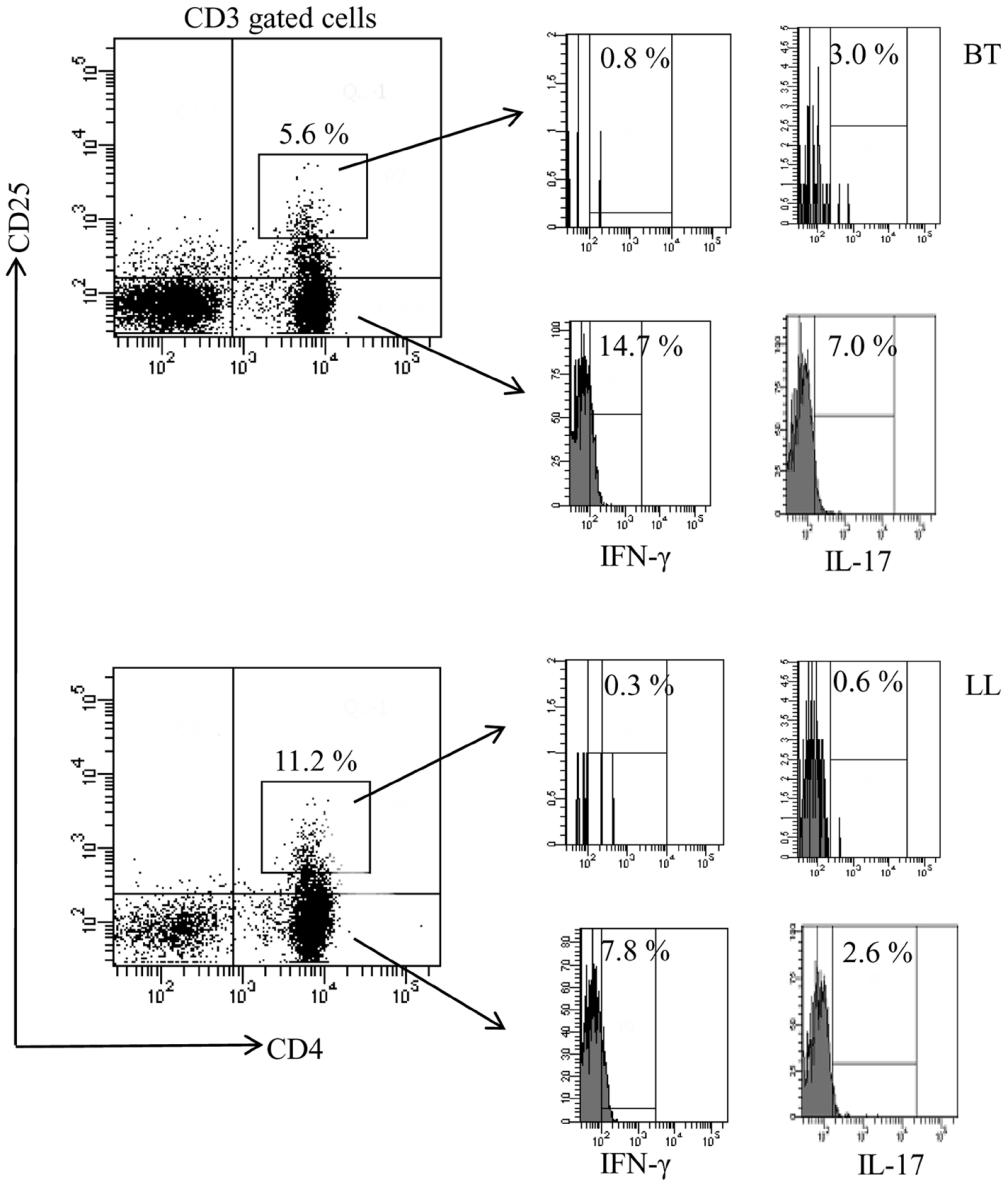
IFN-γ and IL-17 are not produced by CD4^+^CD25^hi^ iTreg cells in lepromatous leprosy. Antigen stimulated PBMC cultures of 3 each of lepromatous and tuberculoid patients were investigated by flowcytometry for the presence of IFN-γ and IL-17 in CD3^+^ gated CD4^+^CD25^hi^ cells. Representative data on each of tuberculoid and lepromatous leprosy patients shows <,1% of IFN-γ+ cells in both leprosy groups. BT showed low percentage of cells with IL-17 which was further decreased in LL. That the negligible cytokine detection was not due to trivial reasons was indicated by CD25-ve population which showed both IFN γ and IL 17, which were as expected higher in tuberculoid as compared to lepromatous subjects. Abbreviations as in legends to Figures 2 and 3.

With a view to understanding the relationship of the above T cells with IFN-γ and IL-17 cytokines reported earlier by us to be associated with BT leprosy [10] we investigated 3 each of BT and LL patients. As may be seen from Figure 7 giving representative data, the CD25^hi^ population of T cells as expected was higher in lepromatous (11–17%) as compared to tuberculoid patients (5–8%). Importantly, IFN-γ was not detectable in the CD25^hi^ population where Mean ± SD of 0.8±0.2 and 0.2±0.1 were observed respectively in tuberculoid and lepromatous patients. That the low percentages were not due to methodological errors was indicated by the detection of IFN-γ-in CD4^+^ CD25^neg^ cells (Mean ± SD of 7.4±1.6 and 4.0±1.8 respectively in tuberculoid and lepromatous patients) confirming that iTregs did not produce this cytokine. However, a small percentage of CD25^hi^ cells were seen to have intracellular IL-17A in (Mean % ± SD: 2.3±0.5) in tuberculoid but not in lepromatous patients (0.63±0.53) with CD4^+^CD25^neg^ population showing respectively Mean% ± SD to be 7.4±1.6 and 4.0±1.8.

#### Other factors associated with CD25^+^FOXP3^+^cells

Other cytokines, transcription factors and signaling molecules reported to be linked to FOXP3 cells were also studied for gene expression using qPCR in both antigen stimulated PBMC and skin lesions. It may seen from Table 3 that expression of IL-25, known to promote FOXP3^+^ cells was significantly increased in lepromatous skin lesions as compared to the tuberculoid type (p<0.01). The latter group showed decreased IL-25 expression as compared to normal skin (p<0.01). In contrast, antigen stimulated PBMC of both types of leprosy showed decreased expression in relation to healthy contacts (p<0.01). IL-27 also showed higher expression in lepromatous as compared to tuberculoid skin (p<0.01) but did not show differences in antigen stimulated PBMC. As expected, IL-2 expression was decreased in lepromatous as compared to tuberculoid (p<0.006) and normal skin (p<0.03) but was not discriminatory in antigen stimulated PBMC of leprosy types. IL-8 studied with a view to investigating trafficking of cells showed increased expression in lesions (p<0.001) but not in antigen induced PBMC cultures of lepromatous leprosy patients. Transcription factor GATA3, STAT3, STAT4, NFkB1, and signaling molecule CD28 showed high expression but failed to discriminate between the clinical types. In summary, skin lesions were more informative with regard to gene expression differences in the leprosy types than the antigen stimulated PBMC for the above markers.

**Table 3:** Gene expression (Mean (ΔCt ± SD) of selected cytokines, signaling molecules and transcription factors associated with FOXP3+Treg and iTreg cells in skin lesions and *M.leprae* stimulated PBMC respectively in leprosy patients and healthy subjects.

ΔCt (Mean ± SD)						
	SKIN			PBMC		
	BT(10)	LL(10)	N(4)	BT(10)	LL(10)	HC (7)
IL-25	12.7±1.8	10.2±2.9^a^	9.2±1.4^c^	8.1±2.5	9.9±3.7	5.6±0.8^d^^,^ e
IL-27	11.9±2.6	8.4±2.4^a^	9.1±1.2	6.7±1.2	6.9±1.3	5.22±0.49^e^
IL-2	9.9±1.2	11.9±2.5^a^	9.5±0.8^b^	6.8±2.4	8.5±4.2	4.3±0.4^e^
IL-8	7.9±1.2	4.2±1.3^a^	8.2±0.6^b^	0.7±1.3	0.64±1.0	0.3±0.7
CD28	12.0±2.0	12.3±3.0^a^	12.0±0.8^b^	6.5±2.9	8.9±3.3	4.3±0.49^d^^,^ e
GATA3	8.2±1.8	8.7±2.3	5.5±1.7^b^	7.0±1.2	7.5±1.7	5.5±1.7
STAT3	3.9±0.7	3.3±1.4	3.1±1.3	2.8±0.7	3.0±0.3	2.6±0.52
STAT4	6.1±1.3	6.7±1.3	9.7±1.1^b^ ^c^	5.1±0.9	5.0±0.8	4.4±0.45
NFκB1	2.6±1.3	2.6±1.0	2.7±1.2	2.5±0.7	2.7±0.4	2.6±0.25

p≤0.05 was considered as significant using Mann Whitney two tailed, non parametric test. Abbreviations: LL: lepromatous leprosy, BT: borderline tuberculoid leprosy. N: skin from normal subjects undergoing cosmetic surgery. HC: healthy household contacts. Figures in parenthesis indicate number of subjects studied. Skin:

^a^ BT vs LL p<0.01 to 0.001;

^b^ LL vs N p<0.02 to 0.01.

^c^ BT vs N p<0.01. PBMC:

^d^ LLvs HC p<0.02,

^e^ BT vs HC p<0.001 by two tailed Mann Whitney test.

p<0.05 was considered significant.

## Discussion

Antigen specific T cell unresponsiveness is the hall mark of lepromatous leprosy and is thought to contribute to chronic disease and the persistence of the leprosy bacillus in the host. The discovery of FOXP3 as a molecular marker of Treg cells has renewed the interest in T cell based mechanisms which dampen effector functions. Experimental models have provided valuable insight into the role of Tregs for tolerance and mucosal immunity including gut infections [16] but their function in other human diseases is not well defined. The impact of FOXP3+ cells in host defence appears to vary with the pathogen as well as the cell manipulations used in *in vivo* and *in vitro* experimental systems [23] Thus, in some viral infections they provided protection [24] in some bacterial infections they are detrimental [25] and in some parasitic infections including malaria, they had no effect [26] Therefore, we investigated the role of CD25^+^FOXP3^+^ Treg cells in the anergic form of lepromatous leprosy. Patients and healthy contacts that had been exposed to leprosy patients were studied due to non availability of animal models that mimic the human clinical types. The present investigation provides evidence for the increase of CD4^+^ CD25^+^ FOXP3^+^ iTreg cells in antigen stimulated PBMC cultures of anergic lepromatous leprosy patients.

Immunostaining of skin lesions showed localization of FOXP3 cells in the granulomas of both types of leprosy with a significant increase in bacilli laden lepromatous lesions. This was supported by the increase in TGF-β and IL-10 producing cells which have been associated with suppression mediated by Treg cells in experimental models [16] and human PBMC [18]. Gene expression studies by qPCR further confirmed the above findings. Our studies in leprosy are consistent with dermal leishmaniasis where T regs were related to the dynamic status of immune responses, appearing in early lesions, decreasing thereafter and reappearing in chronic lesions [27]. Differing patterns were observed in post kala azar dermal leishmaniasis lesions where patients from India [28] but not from Sudan [29] showed increased Tregs cells in the skin. To understand the nature and development of FOXP3^+^cells in leprosy, we combined gene expression for a wide variety of markers and transcription markers associated with Treg cells with flow cytometry for phenotypic characterization and ELISA for relevant cytokines in *in vitro* antigen stimulated PBMC cultures Increase in gene expression of Treg signatures, *FOXP3*, *TGF-β* and *IL-10* was observed in lepromatous as compared to tuberculoid patients.

Flow cytometry analysis also showed increase in CD4+CD25^+^FOXP3^+^ cells in unstimulated basal cultures suggesting either presence of ‘natural tT reg’ or *in vivo* induced Tregs during the course of the disease. Further increase in *in vitro* cultures of antigen but not PHA stimulated PBMC of lepromatous leprosy patients indicated induction of iTregs. Both percentages of these cells as well as MFI values showed increase in lepromatous as compared to tuberculoid subjects for FOXP3 and TGFβ in the CD4+CD25+ T cell populations. Moreover ELISA showed increased levels of TGF-β (p<0.0003) in the culture supernatants of stimulated PBMC from lepromatous as compared to tuberculoid subjects. IL-10 was also increased in the culture supernatants of lepromatous as compared to tuberculoid leprosy patients (p<0.02) In general, increase in FOXP3+ cells in leprosy is in agreement with other studies in leprosy; however there are differences in the nature of cytokine associated with them. Increased association of TGF-β after antigen stimulation [30] as well as increase in a subset of Treg cells with IL-10 in anti-CD3 and anti-CD28 stimulated PBMC was observed in lepromatous patients [31]. Our results are in conformity with those of Palermo et al [32] wherein PBMC of Brazilian lepromatous patients were reported to show increase in CD25^+^ FOXP3^+^cells but differ in the associated inhibitory cytokine. Whereas association of IL-10 was observed by them we found iTreg association with TGFβ [32]. Varied results were also reported where increase in FOXP3^+^ cells was associated with tuberculoid leprosy using flow cytometry analysis [33]. Treg increase both in the blood and in pleural fluid was shown in patients with active tuberculosis, whose causative pathogen is similar to that of leprosy [34].

That TGF-β was produced by CD4^+^CD25^+^ FOXP3^+^ population was confirmed by flowcytometry on antigen stimulated PBMC. Intracellular TGF-β required phosphorylation of STAT5A. Though high gene expression of STAT5A was observed both in the skin and in PBMC, no significant differences were observed in the clinical groups. Importantly, phosphorylation appeared to be a pre-requisite as p-STAT5A was higher in CD25^hi^ FOXP3+ cells of lepromatous patients and >90% of TGF-β producing cells was associated with phosphorylated STAT5A. This transcription factor after activation by IL-2 has been shown to bind to *FOXP3* gene and cooperate with STAT3A for FOXP3 induction [35]. Activation of STAT5 has divergent effects on T cell subsets, leading to expansion of CD8^+^ memory cells and development of CD4^+^CD25^+^ regulatory T cells [36]. CD8^+^ lineage with CD25^+^FOXP3^+^ were also increased in lepromatous leprosy subjects but they did not show intracellular TGF-β.

Differences between the two clinical types of leprosy were only observed with TGF-β producing CD4+T cells which had CD25^+^FOXP3^+^ phenotype (p<0.002) and not with FOXP3^+^ cells alone or where CD25 was absent. It is of interest that FOXP3^+^ iTreg cells were higher in healthy contacts (p<0.001) as compared to tuberculoid leprosy suggesting that dampening of immune responses during early stages of infection may help in protection from clinical disease akin to the dynamic state noted in dermal leishmaniasis [25]. In most experimental models and in T cell clones CD25^hi^ cells have been incriminated for suppressive ability [16]. Though our studies have not formally established that CD25^+^ FOXP3^+^cells exerted suppression, nevertheless, they seem to be associated with the T cell anergy in leprosy as both CD25^hi^ (p<0.03) and CD25^low^ (p<0.004) FOXP3^+^ cells showed significant increases in the anergic lepromatous as compared to the limited form of tuberculoid leprosy.

Furthermore, whereas ELISA on culture supernatants of antigen stimulated PBMC showed IFN-γ and IL-17 to be increased in tuberculoid and not in lepromatous leprosy, Treg cells did not show intracellular IFN-γ in either type of leprosy. The latter is in agreement with the down regulation of this cytokine observed by Palermo et al [32]. However, flow cytometry on antigen stimulated PBMC detected a small percentage of IL-17A producing cells in tuberculoid patients. The latter feature is of interest as we had shown recently that Th17 cells were associated more with tuberculoid leprosy and the non polarized Th0 phenotypes in both types of leprosy [10]. Further studies are required to define the relationship of Th17 and iTreg cells. Kumar et al [31] showed that TGF-β led to increased phosphorylation of SMAD3, NFATC and facilitation of FOXP3 expression with low ubiquitination adding to the stability and suppressive potential of the Treg cells in leprosy.

The peripheral population of Tregs studied by us has features of both natural (n/tTreg) and iTreg populations. Unstimulated basal PBMC showing CD4+CD25^+^FOXP3^+^ cells may indicate nTreg. Alternatively they may belong to the *in vivo* generated Tregs of an ongoing natural immune response in the untreated leprosy patients. Unstimulated *ex vivo* PBMC of tuberculoid subjects showed low numbers (1–15%) whereas the generalized lepromatous patients showed <30% of the Tregs with intracellular TGF-β in the unstimulated PBMC. Though there is an overlap between nTreg and iTreg in certain situations with regard to this cytokine, consensus exists for its association with iTregs. On antigen stimulation this population of cells increased further in the lepromatous patients, which may be related to the expansion of pre-existing iTreg population. The stimulated PBMC in both leprosy types showed many fold increase in TGF-β.

Our studies also showed some features that need further investigation. Of interest was the sequential decrease in MFI of FOXP3 in CD25^hi^, CD25^low^ and CD25^neg^ cells. It has been reported that CD25^neg^ cells show transient expression of low FOXP3 [17], [18] which is in agreement with our study in leprosy. Such cells were reported to be functionally non regulatory/suppressive [17], [18]. Further studies are required to formally establish the functional nature of such FOXP3 cells in leprosy.

IL-2 considered to be critical for both types of Treg cells, showed decreased expression in both types of leprosy as compared to healthy subjects in PBMC and between the two leprosy types in the skin. Furthermore, earlier studies from several groups, including ours had shown marked reduction of this cytokine in lepromatous patients [22]. It has been suggested that T cells do not suppress the initial activation of CD4^+^CD25^neg^ T cells but influence inhibition by the production of IL-2 by the effector cells which results in expansion of the Tregs and subsequent suppressor function [37]. Thus the discrepancy noted in our studies may be related to time kinetics of early antigen interaction which is difficult to capture in a disease which has a long incubation period. CD28 co-stimulatory signals considered to be essential for differentiation of nTreg/iTregs and expression of FOXP3 independently of IL-2 [16]. Expression of CD28 did not discriminate the leprosy types. GATA3 shown to control FOXP3^+^ regulatory function in dermal and gut inflammation in murine models [38] showed a lack of association with the clinical groups in antigen stimulated PBMC. This was puzzling as GATA3 is a Th2 transcription factor and many lepromatous patients show a Th2 polarization state. In our study, GATA3 showed decrease in the dermal lesions of lepromatous leprosy as compared to normal skin. In *M.tuberculosis* infection, T-bet expressing Treg cells and effector cells have been shown to expand under Th1 conditions [39] whereas Tregs with GATA-3 were seen under Th2 conditions [40].These differences may be related as indicated earlier [23] to differences between pathogens, experimental and human models of disease, sites of inflammation, as well as differences between stimulation of naïve T cells as compared to recall responses studied by us.

Importantly, our studies show that the antigen specific T cell anergy and cytokine dysregulation associated with lepromatous leprosy may be linked to the increase in TGF-β producing iTreg population belonging to a suppressive lineage of T cells. The role of bacillary load (BI) on the evolution and maintenance of this population requires studies on lepromatous leprosy patients after negligible BI is achieved. This proves to be a logistic problem in public health as in the current regimen patients are released from treatment after 1 year when their BI is still positive since the BI reduction is at the rate of 1 log per year.

Our earlier studies on lepromatous patients had incriminated monocytes/macrophage lineage and their soluble factors containing prostaglandin E2, leukotrines and throboxanes in the inhibition of *in vitro* T cell proliferation in tuberculoid subjects [6]. Recent studies have indicated that prostaglandin E2 induces *FOXP3* gene expression and iTreg function in human CD4^+^ T cells [41], [42] which is compatible with our findings. Thus soluble factors released by bacilli laden monocytes/macrophage may play a role in inducing Treg cell function in lepromatous leprosy thereby resulting in the iTreg mediated antigen specific unresponsiveness associated with this disease.

## Supporting Information

Figure S1 Lepromatous leprosy patients (LL) show low levels of IFN-γ in *M.leprae* stimulated PBMC cultures as compared to tuberculoid patients (BT) in ELISA (p<0.001, two tailed Mann Whitney). All patients investigated for gene expression and flow cytometry analysis were evaluated for antigen specific responses using IFN-γ as a surrogate marker of T cell responses. A. Scatterdiagram of Mean pg/ml ± SD of IFN-γ in duplicate samples of culture supernatants of antigen stimulated PBMC tuberculoid (768.5±1133) and lepromatous patients (57.2±22.6) by ELISA.**B**. Flow cytometry analysis of one each of BT and LL patients. Stimulated PBMC were gated for live lymphocytes as in upper left hand panel, then CD3+ cells in middle panel were analyzed for IFN-γ in both CD4+ and CD8+ populations (right hand panel) as indicated by arrows in both types of leprosy types. Numbers show the percentage of positive cells. Flow cytometry strategy and validation of antibodies is given in Figure S2.
(TIF)

Figure S2 Optimization of staining, manual gating, isotype controls and combined dot plots used in multi color flowcytometry analysis for identification of CD4^+^FOXP3^+^ iTregs in a representative tuberculoid leprosy patient. Total number of cells were kept constant at 0.5×10^6^. After selecting for singlets, lymphocytes were further selected using SSC and FSC parameters, **A**. panel showing FMO (fluorescence minus one) and stained cells for each T cell marker. **B**. CD3^+^ lymphocytes were derived from CD3^+^ versus SSC. CD4^+^ and CD8^+^ cells were derived from the CD3^+^ population. Tregs were identified by CD25^+^ versus FOXP3^+^ from both CD4^+^ and CD8^+^ T cells using dot blots. **C**. Isotype controls used for FITC (fluoresceine isothyocyanate) APC (allophycocyanin) and PE (phycoerythrin) labeled antibodies.
(TIF)

Figure S3 PHA (phytohemagglutinin) stimulated PBMC show increase in percentage of CD 4^+^CD25^+^ FOXP3^+^ T cells in leprosy patients as compared to healthy subjects by flow cytometry analysis which was further confirmed by Mean Fluorescence Intensity (MFI). Though percentage of TGF-β bearing cells of the above lineage did not discriminate between the clinical groups, MFI showed statistically significant differences between tuberculoid and healthy subjects as shown in the figure Abbreviations: BT: borderline tuberculoid leprosy, LL: lepromatous leprosy; HC: healthy contacts. ( ) Parenthesis indicates number of subjects.
(TIF)
