# Supplementary figures and images for: Increase in TGF-β Secreting CD4+CD25+ FOXP3+ T Regulatory Cells in Anergic Lepromatous Leprosy Patients
Source: PLoS Negl Trop Dis. 2014 Jan 16;8(1):e2639. doi: 10.1371/journal.pntd.0002639 (PMC3894184; doi:10.1371/journal.pntd.0002639)

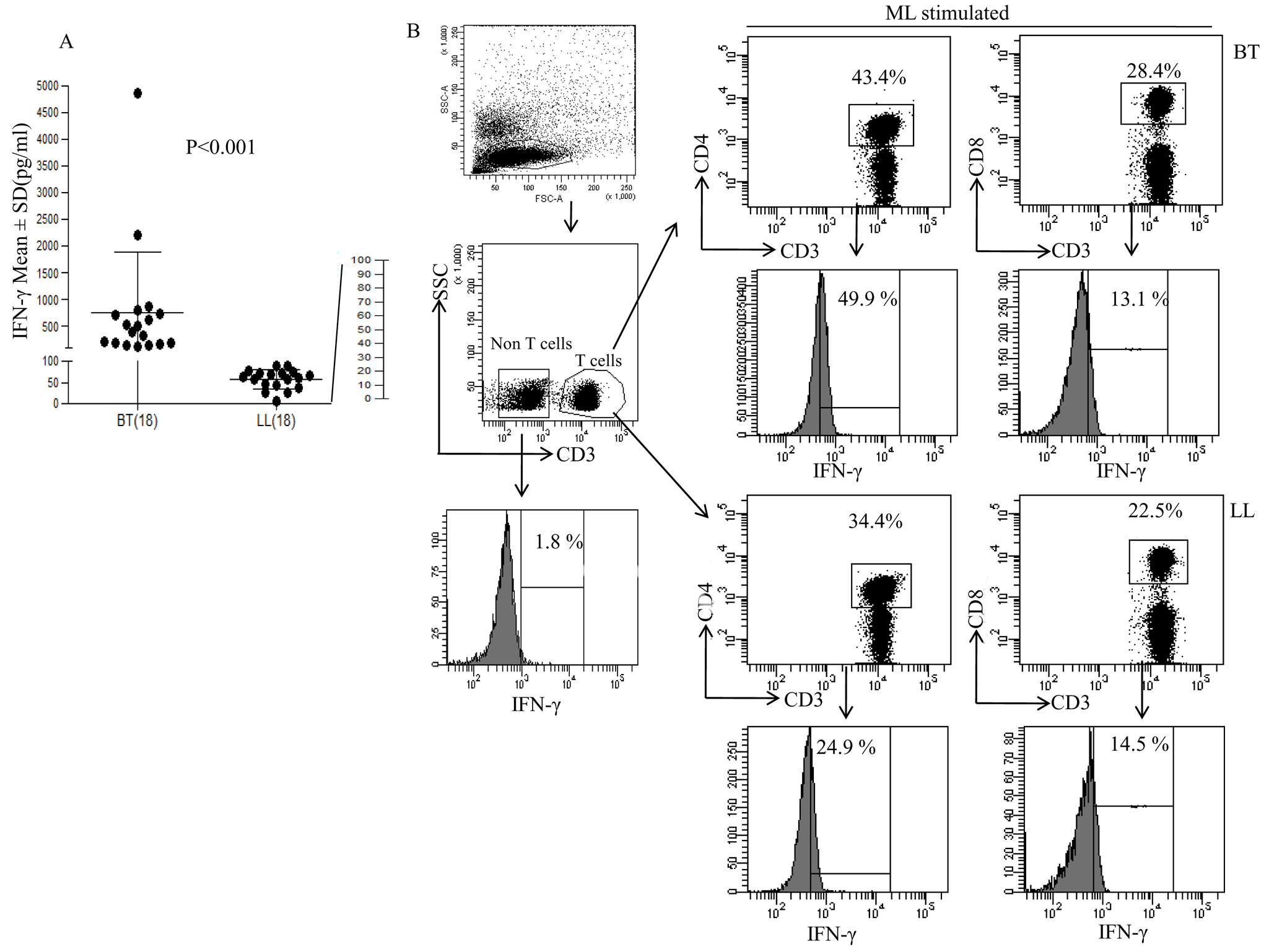

Supplement: Figure S1 — Lepromatous leprosy patients (LL) show low levels of IFN-γ in M.leprae stimulated PBMC cultures as compared to tuberculoid patients (BT) in ELISA (p<0.001, two tailed Mann Whitney). All patients investigated for gene expression and flow cytometry analysis were evaluated for antigen specific responses using IFN-γ as a surrogate marker of T cell responses. A. Scatterdiagram of Mean pg/ml ± SD of IFN-γ in duplicate samples of culture supernatants of antigen stimulated PBMC tuberculoid (768.5±1133) and lepromatous patients (57.2±22.6) by ELISA.B. Flow cytometry analysis of one each of BT and LL patients. Stimulated PBMC were gated for live lymphocytes as in upper left hand panel, then CD3+ cells in middle panel were analyzed for IFN-γ in both CD4+ and CD8+ populations (right hand panel) as indicated by arrows in both types of leprosy types. Numbers show the percentage of positive cells. Flow cytometry strategy and validation of antibodies is given in Figure S2. (TIF) [file pntd.0002639.s001.tif]

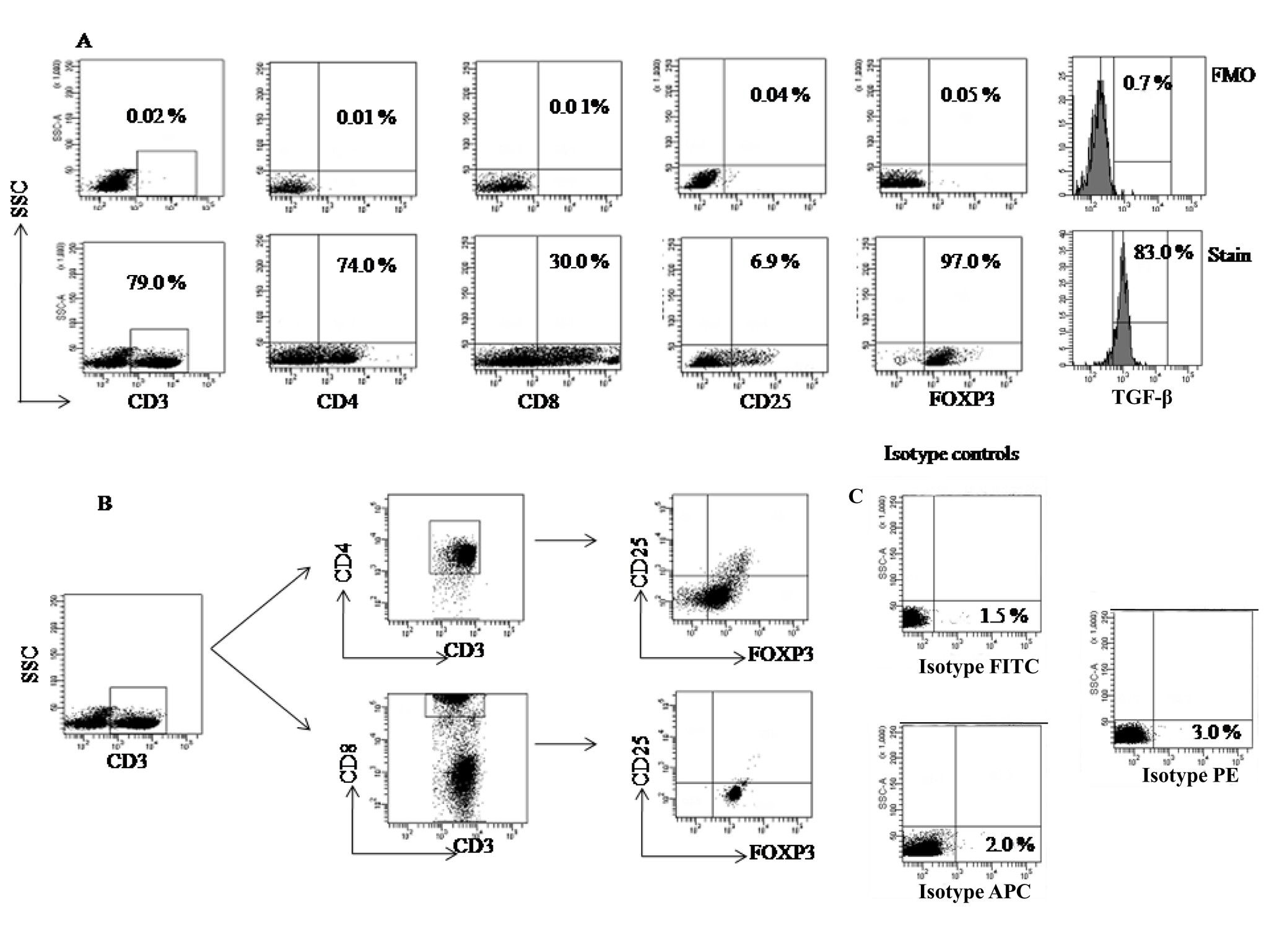

Supplement: Figure S2 — Optimization of staining, manual gating, isotype controls and combined dot plots used in multi color flowcytometry analysis for identification of CD4+FOXP3+ iTregs in a representative tuberculoid leprosy patient. Total number of cells were kept constant at 0.5×10^6^. After selecting for singlets, lymphocytes were further selected using SSC and FSC parameters, A. panel showing FMO (fluorescence minus one) and stained cells for each T cell marker. B. CD3+ lymphocytes were derived from CD3+ versus SSC. CD4+ and CD8+ cells were derived from the CD3+ population. Tregs were identified by CD25+ versus FOXP3+ from both CD4+ and CD8+ T cells using dot blots. C. Isotype controls used for FITC (fluoresceine isothyocyanate) APC (allophycocyanin) and PE (phycoerythrin) labeled antibodies. (TIF) [file pntd.0002639.s002.tif]

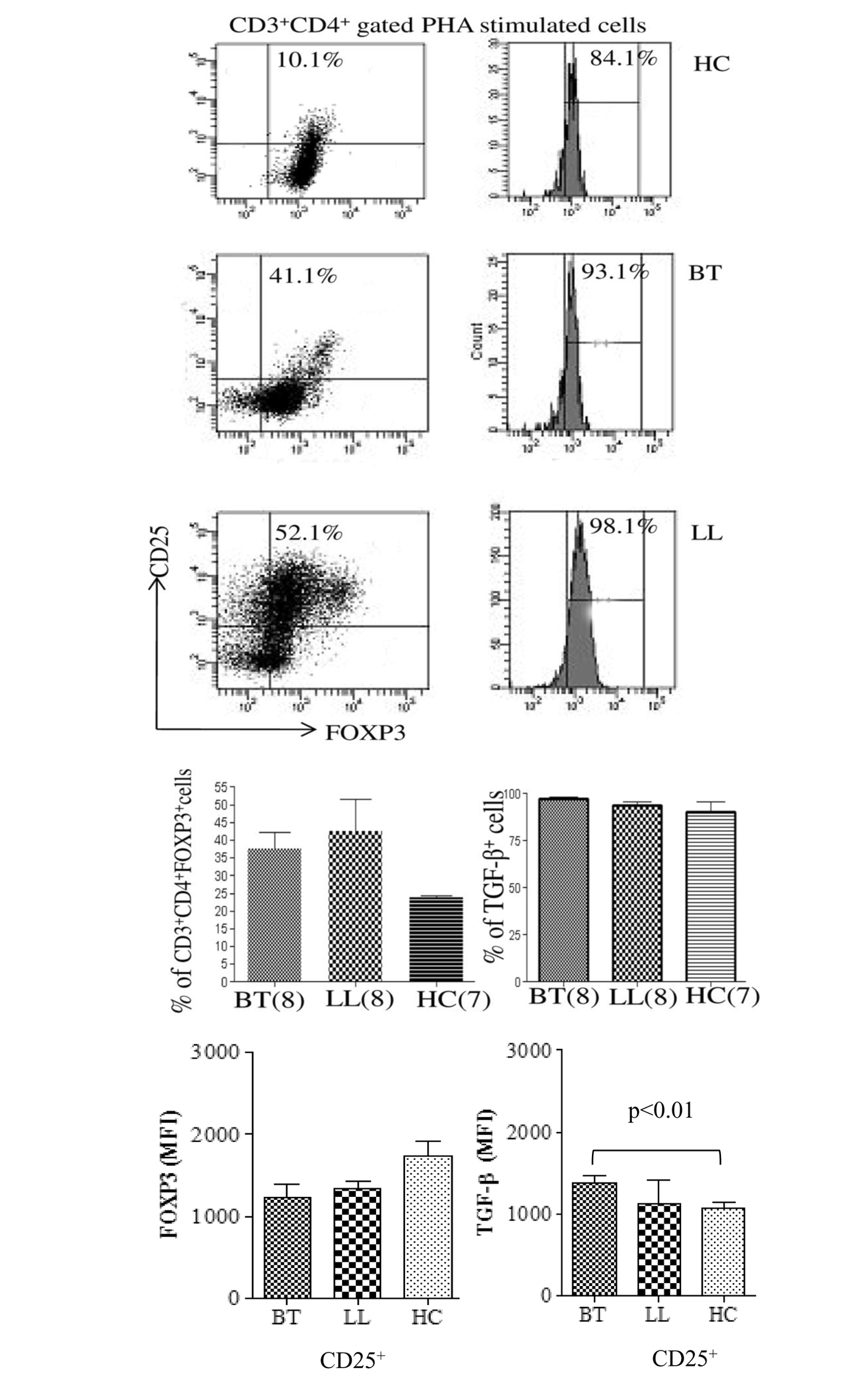

Supplement: Figure S3 — PHA (phytohemagglutinin) stimulated PBMC show increase in percentage of CD 4+CD25+ FOXP3+ T cells in leprosy patients as compared to healthy subjects by flow cytometry analysis which was further confirmed by Mean Fluorescence Intensity (MFI). Though percentage of TGF-β bearing cells of the above lineage did not discriminate between the clinical groups, MFI showed statistically significant differences between tuberculoid and healthy subjects as shown in the figure Abbreviations: BT: borderline tuberculoid leprosy, LL: lepromatous leprosy; HC: healthy contacts. ( ) Parenthesis indicates number of subjects. (TIF) [file pntd.0002639.s003.tif]
